# Joint inflammation related citrullination of functional arginines in extracellular proteins

**DOI:** 10.1038/s41598-017-08597-4

**Published:** 2017-08-15

**Authors:** Kalle H. Sipilä, Vipin Ranga, Pekka Rappu, Markku Mali, Laura Pirilä, Ilona Heino, Johanna Jokinen, Jarmo Käpylä, Mark S. Johnson, Jyrki Heino

**Affiliations:** 1 Department of Biochemistry, University of Turku, Turku, Finland; 2 Structural Bioinformatics Laboratory, Biochemistry, Faculty of Science and Engineering, Åbo Akademi University, Turku, Finland; 3 Turku University Hospital, Division of Medicine, Department of Rheumatology, and University of Turku, Turku, Finland

## Abstract

We report the extent, specific sites and structural requirements of joint inflammation related citrullination in extracellular proteins. A total of 40 synovial fluid samples derived from chronically inflamed human joints were analysed by heparin-agarose fractionation and LC-MS/MS. Citrullination of 55 arginines in extracellular proteins was detected. Importantly, 20% of the sites have a characterized function related to the hallmarks of destructive joint inflammation. E.g. four arginine residues, shown here to be citrullinated, are also affected by mutations in inherited diseases causing haemolysis or blood clotting dysfunction. Citrullination of integrin ligands was selected for further studies since fibronectin R234 in isoDGR was among the most frequently citrullinated arginines in synovial fluid. Assays with synovial fibroblasts and integrin αVβ3 indicated decreased affinity to the enzymatically citrullinated integrin binding sites. To conclude, our data indicate that in inflamed joints extensive citrullination affects the functional arginine residues in extracellular proteins.

## Introduction

The composition and organization of the extracellular matrix (ECM) are the major determinants of tissue integrity and cellular homeostasis. Adhesion receptors, especially the members of the integrin family, mediate the anchorage of cells to ECM and orchestrate chemical and mechanical signal transduction^1^. Post-translational modifications (PTMs) generated by interstitial enzymes or non-enzymatic reactions further increase the structural and functional diversity of ECM proteins and fibrils. In normal tissues, PTMs may lead to covalent intra- and intermolecular bonds and modify the integrity of ECM. For example, the enzyme lysine 6-oxidase (lysyl oxidase, LOX) increases ECM stiffness by creating collagen cross-links^2^. The increasing number of documented, active extracellular protein kinases and phosphatases suggests that the regulation of protein function outside the cells by PTMs may be even more extensive than previously considered^3^.

Frequently, PTMs of ECM proteins have been linked to pathological processes seen in various human diseases: In cancer LOX activity may significantly promote tumour progression, while in diabetes and during ageing non-enzymatic glycation may contribute to blood vessel related complications. Furthermore, non-enzymatic carbamylation has been connected to chronic kidney disease^4, 5^.

Furthermore, rheumatoid arthritis (RA) related autoantibodies that recognize epitopes harbouring in extracellular proteins, such as collagen II and fibrinogen, have been a target of extensive research. Such epitopes can contain PTMs, for example, carbamylated lysine (homocitrulline) or deimidated arginine (citrulline)^6^. The presence of anti-citrulline protein antibodies (ACPA) is a well-established and highly specific biomarker for RA^7^. Autoantibodies recognizing carbamylated proteins are predictive for a more severe clinical outcome^8^. In addition, autoantibodies that recognize oxidized protein epitopes, caused by reactive oxygen species, have been reported^9^.

In general it is an emerging question, in which magnitude PTMs in extracellular proteins also modify cellular interactions and consequently cell behaviour. *In vitro* experiments, often based on the exposure of proteins to enzymes or chemical agents, have indicated that PTMs can modify the function of integrin binding motifs^4^. However, the extent, specificity and underlying mechanisms of this phenomenon are mostly unknown. Here, we performed liquid chromatography-tandem mass spectrometry (LC-MS/MS) based analysis of 40 synovial fluid samples derived from inflammatory arthritis patients. The only extracellular PTM that was detected to correlate with the activity of inflammation was citrullination. Many of the arginine residues, that here were found to be citrullinated, have previously been shown to participate in biologically and pathophysiologically important protein–protein interactions. Importantly, in three cases a citrullinated arginine was a critical residue in an integrin recognition motif, and the citrullination modulated cell adhesion mechanisms.

## Results

### Functional motifs in extracellular proteins are citrullinated in chronic inflammation

**Figure 1:**
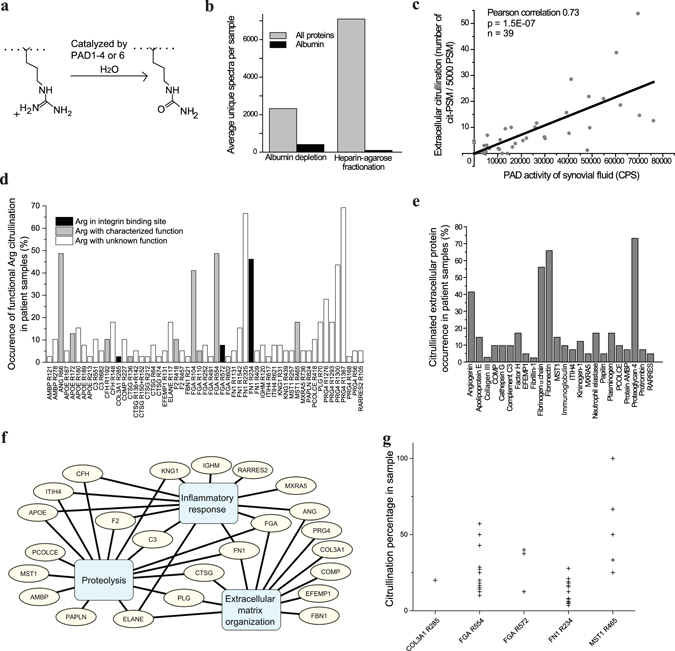
The analysis of citrullination in human synovial fluid samples. (**a**) Illustration of protein citrullination by PAD enzyme. (**b**) Number of detected unique spectra by mass spectrometry in albumin depleted or heparin fractionated synovial fluid samples (n = 11). (**c**) Correlation of extracellular citrullination (mass spectrometry, MS) and PAD activity (enzyme assay). (**d**) Detected citrullinated sites of extracellular proteins in synovial fluid samples. (**e**) The citrullinated extracellular proteins identified in synovial fluids (n = 40). The frequency (%) of samples containing the given citrullinated protein is shown. (**f**) The relationships of the extracellular citrullinated proteins to selected biological processes according to the GO Database 6/2014 release^45^ appended with current knowledge of the biological role of angiogenin^46–48^, MXRA5^49^, PRG4^50^ and EFEMP1^51^. IGHM, which is a subunit of IgM is connected to inflammatory response. (**g**) Citrullination levels of the arginines with a characterized function in individual samples. Only the arginines present in both citrullinated and non-citrullinated peptides were included.

In order to study extracellular citrullination (Fig. 1a), we performed heparin fractionation of 40 synovial fluid samples derived from 31 inflammatory arthritis patients followed by in-solution trypsin digestion and LC-MS/MS analysis. Heparin-agarose fractionation allowed us to effectively remove hyaluronate and albumin as well as to enrich the heparin binding extracellular proteins (Fig. 1b).

The correlation between PAD activity in synovial fluid, measured by an enzyme assay, and the amount of citrullinated peptide-spectrum-matches (PSM), normalized to the total PSM number in the sample, was very strongly positive (Pearson’s r = 0.73, p-value = 1.5E-7) (Fig. 1c). We could identify 55 arginine residues in 24 different extracellular proteins that are targets of extracellular citrullination (Fig. 1d,e, Supplementary Table 1). All citrullinated arginine residues with their detection frequency are presented in Fig. 1d. The most frequently citrullinated arginine residues could be found in about 60% of the samples (Fig. 1d). Angiogenin, fibrinogen, fibronectin (FN) and proteoglycan 4 were the most frequently citrullinated proteins (Fig. 1e). Pathway analysis suggested that the proteins that become citrullinated are often related to the hallmarks of destructive joint inflammation: immune response, proteolysis and tissue remodeling^10^ (Fig. 1f). In general more than 20% of the arginine residues that were citrullinated have a known function in ligand–receptor recognition, enzymatic activity, proteolytic cleavage or other molecular interactions (Supplementary Table 2), which also suggests that extracellular citrullination can directly affect the pathogenesis of joint inflammation. On the basis of ratios between citrullinated and detectable non-citrullinated peptides, we estimated that the citrullination level of functional arginines varied between 3 to 100% depending on the site, the protein and the overall citrullination level in the sample (Fig. 1g).

### Solvent exposed arginine residues are primary targets of PADs

**Figure 2:**
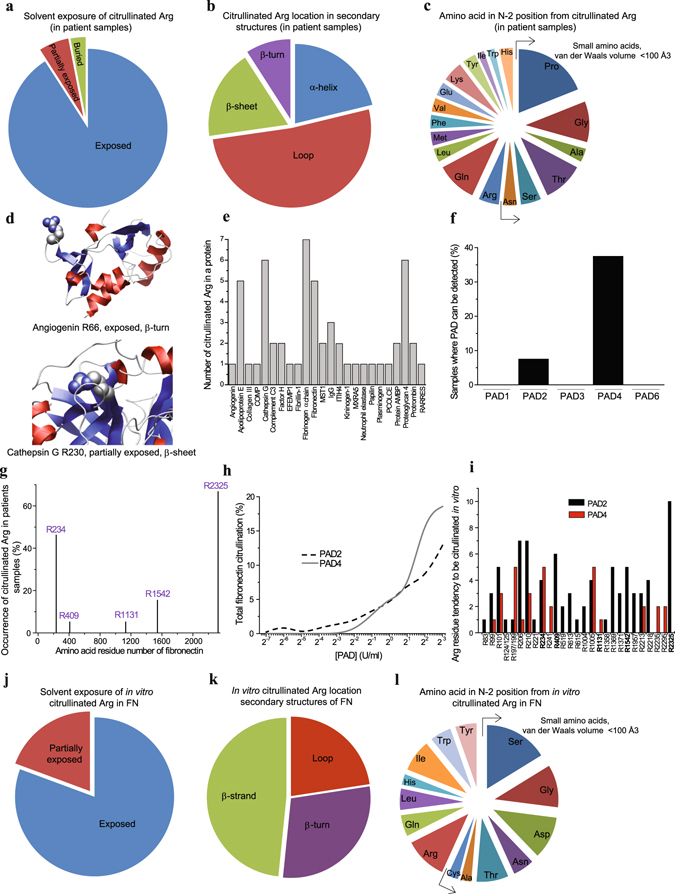
Extracellular citrullination is a consequence of non-selective citrullination targeted to exposed arginine side chains. The arginine residues corresponding to citrullinated arginines: (**a**) Solvent exposure in known 3D structures. (**b**) Frequency of citrullinated arginine residue location within a protein secondary structure type. (**c**) Frequency of amino acid types at position N-2 in the sequence relative to citrullinated arginine. Examples for arginine location in 3D structures: (**d**) R66 of angiogenin and R230 of cathepsin G. (**e**) The number of citrullination sites in single proteins identified in synovial fluid in inflammation. (**f**) The frequency of PAD2 and PAD4 detection in synovial fluid samples by mass spectrometry. (**g**) The citrullination sites in fibronectin and the frequency of citrullination among synovial fluid samples. (**h**) Fibronectin citrullination by PAD2 and PAD4 *in vitro* detected by mass spectrometry. (**i**) Fibronectin *in vitro* citrullination sites. The value of the arginine tendency to be citrullinated is the number of samples in which the citrullinated arginine can be detected in different PAD concentrations (7.8 mU/ml–8 U/ml). (**j**–**l**) For arginine residues corresponding to *in vitro* citrullinated arginines in fibronectin: (**j**) Solvent exposure in known 3D structures. (**k**) Frequency of *in vitro* citrullinated arginine residue location within a protein secondary structure type (note: fibronectin does not contain α helices). (**l**) Frequency of amino acid types at position N-2 in the sequence relative to *in vitro* citrullinated arginine.

In order to study whether the citrullination of arginine residues, detected in human synovial fluid, is a consequence of selective or nonselective enzymatic process for integrin ligands and extracellular proteins in general, we analysed the structural characteristics of the citrullination sites in the X-ray structures of the corresponding proteins (Fig. 2; Supplementary Table 3). The vast majority of citrullinated arginine residues seemed to be solvent exposed (Fig. 2a), while there does not appear to be any distinctive consensus sequence or structural motif that would lead to the citrullination of arginine residues (Fig. 2a–c). Figure 2d shows the models of R66 in angiogenin and R230 in cathepsin G, which are both located on a β-turn. In few cases only the residues are partially or completely buried, e.g. R230 in cathepsin G. R230 is located on β-sheet and has a partially exposed side chain (Fig. 2d). Based on the X-ray structure of PAD4 in complex with the histone N-terminal tail, a previous report claims that two sequential residues on either side of the arginine that is citrullinated are bound within the active/binding site of the PAD enzyme^11^. The entire arginine side chain penetrated deeply into the active site, and residues N-1, N + 1 and N + 2 (numbering relative to arginine) were bound to the PAD enzyme only through main-chain interactions, providing no specificity related to the type of side chain involved. The N-2 position was suggested to provide some specificity. Small, polar residues like serine and threonine (PDB ID: 2DEY and 2DEW)^11^ can form hydrogen bonds with Q346 and D344 of the enzyme PAD4. Consequently the authors proposed, based on the X-ray structure, that arginine residues citrullinated by PAD4 would be located on a β-turn-like loop conformation, and that there would be a preference for a small residue at the N-2 position^11^. However, the data presented here, and in details in Supplementary Table 3, clearly show that while the loop secondary structure is preferred (Fig. 2b), probably due to inherent solvent exposure (Fig. 2a), arginines that are citrullinated are found on α-helices, β-strands and β-turns (Fig. 2b). While the N-2 position is predominated by proline and glycine, the residues also found with high frequency in loops and β-turns, it is clear that any of the 20 amino acids can be at the N-2 position (Fig. 2c). Together these data show that arginine residues that are citrullinated must be or become solvent exposed, but that they can occur within any secondary structure and there are no residue preferences. The number of citrullination sites in single proteins identified in synovial fluid are shown in Fig. 2e. Citrullination of up to seven different arginine residues per protein could be detected (Fig. 2e).

PAD2 and PAD4 were selected for *in vitro* studies since they are the only PAD isotypes that can be detected in synovial fluid (Fig. 2f). In these experiments we compared the synovial fluid (Fig. 2g) and *in vitro* citrullination patterns of FN. *In vitro* both PAD2 and PAD4 citrullinated FN (Fig. 2h) and gave unique but still partially overlapping citrullination patterns of arginine residues (Fig. 2i). Despite the fact that many arginine residues could be citrullinated, PAD2 and PAD4 citrullinated some residues more frequently than the others. Based on our observations, the arginine residues in FN that were targets of citrullination in synovial fluid (Fig. 2g), could also be deiminated by PADs *in vitro* (Fig. 2i). However, many other residues were citrullinated *in vitro* with similar efficacy despite the fact that their citrullination was not detected in synovial fluid. Thus, based on simple *in vitro* experiments only, the prediction of the citrullination pattern in human samples is very difficult or impossible. More intense fractionation of synovial fluid might have helped in the detection of more citrullinated arginine residues. However, our data stress the importance of direct analysis of patient derived samples before definitive conclusions.

In FN, in accordance with the synovial fluid samples, the effectively *in vitro* deiminated arginine residues are highly exposed to solvent (Fig. 2j). FN is composed of β-strands, β-turns and loops, but lacks α helical folds. About half of the citrullinated arginine residues are found on β-strands (Fig. 2k), which is again consistent with the observation that no particular secondary structure is a prerequisite for citrullination by PAD enzymes. In FN small and large side chains can be found at the N-2 position (Fig. 2l). Serine is most often in that position, but the number of observations is low. Thus, also the FN data speak against the idea that the four residues that surround the arginine could form any distinct motif that mediates the binding to the active site of the PAD enzymes. Sequence and structural features associated with FN citrullination are listed in Supplementary Table 4.

### The extent of citrullination in human synovial fluid correlates to the intensity of inflammation

**Figure 3:**
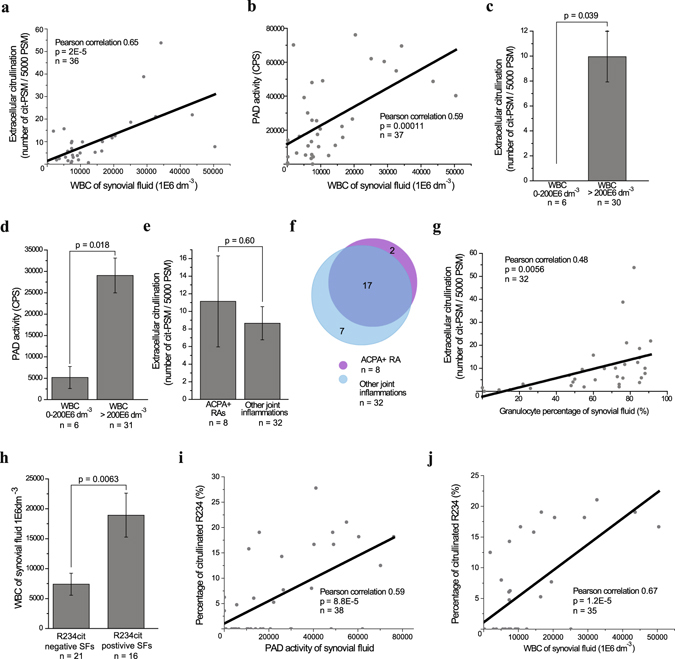
Extracellular citrullination associates with inflammation markers. (**a**) Correlation of extracellular citrullination (mass spectrometry, MS) and white blood count (WBC). (**b**) Correlation of PAD activity (enzyme assay) and WBC. (**c**) The level of extracellular citrullination (mass spectrometry) in inflammatory (WBC over 200 × 10^6^ dm^−3^) and non-inflammatory synovial fluids. (**d**) PAD activity (enzyme assay) in inflammatory (WBC over 200 × 10^6^ dm^−3^) and non-inflammatory synovial fluids. (**e**) Level of extracellular citrullination (mass spectrometry) in anti-citrulline antibody positive and negative RAs. (**f**) Number of identified citrullinated extracellular proteins in anti-citrulline antibody positive and negative RAs. (**g**) Correlation between extracellular citrullination and the relative amount of granulocytes among white blood cells. The number of samples varies because in some cases the university hospital did not routinely perform standard assays. (**h**) The WBC in the synovial fluid samples in which the citrullination of R234 in fibronectin, the known integrin binding site, can be detected. (**i**) The correlation between the citrullination level of R234 in fibronectin and PAD activity. (**j**) The correlation between the citrullination level of R234 in fibronectin and WBC. P-values were calculated with two-tailed Student’s t-test. Error bars show SEM.

The global analysis of deamidation (Supplementary Fig. 1a,b), phosphorylation (Supplementary Fig. 1c), proline hydroxylation (Supplementary Fig. 1d), carbamylation (Supplementary Fig. 1e), acetylation (Supplementary Fig. 1f), and citrullination (Fig. 3a) revealed that citrullination was the only extracellular PTM significantly correlating (Pearson correlation 0.65, p = 2E-5; 1.4E-4 after Bonferroni correction for multiple comparisons) with the number of leukocytes in the synovial fluid (white blood cell count, WBC).

In addition, PAD activity correlated to the WBC (Fig. 3b). Furthermore, when the synovial fluid was considered to be non-inflammatory, i.e. WBC was under 200 × 10^6^/dm^3^, we could not detect general citrullination of extracellular proteins. Accordingly PAD activity was significantly lower (Fig. 3c,d). No significant difference could be detected when citrulline antibody (ACPA) positive rheumatoid arthritis (RA) patients were compared to individuals with other joint inflammations, including psoriatic arthritis, juvenile polyarthritis, and gout (Fig. 3e, Supplementary Fig. 2). Nineteen extracellular proteins were identified as citrullinated in the synovial fluids of the ACPA positive patients (n = 8) and the citrullination of 24 extracellular proteins was evident in the synovial fluids of other joint inflammation patients (n = 31). Seventeen proteins were overlapping between the patient groups (Fig. 3f). In addition, the relative number of granulocytes weakly correlated with the amount of extracellular protein citrullination (Fig. 3g). Likewise the extracellular citrullination in general, citrullination of R234 in FN strongly correlated with leukocyte infiltration into the joint (Fig. 3h–j).

### Citrullination affects integrin recognition motifs

**Figure 4:**
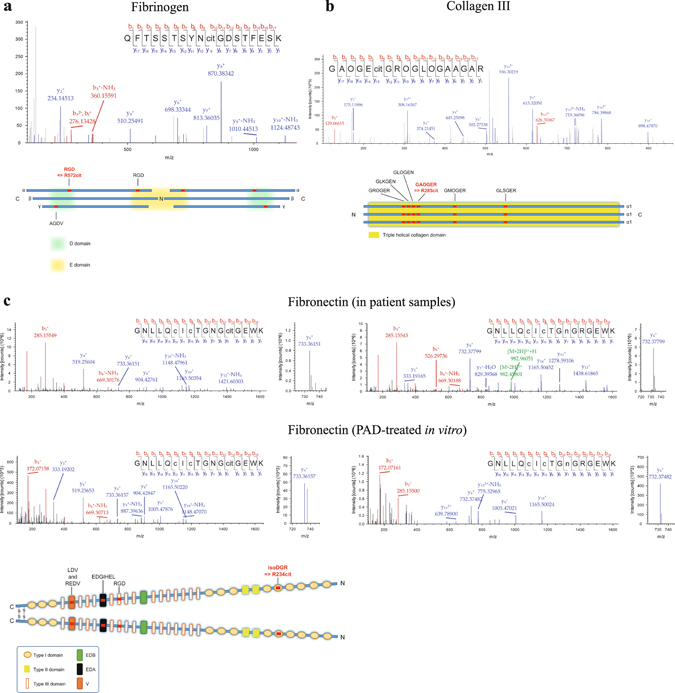
Citrullination of three integrin binding motifs, isoDGR in FN, RGD in fibrinogen and GAOGER in collagen III can be detected in human synovial fluid in inflammation. (**a**) Tandem MS spectrum matching to a fibrinogen alpha derived tryptic peptide containing a citrullinated RGD site. Bottom, schematic illustration of citrullinated integrin binding site in fibrinogen. Integrin binding sites are labelled red. (**b**) Tandem mass spectrum matching to a tryptic peptide containing the integrin binding motif GAOGER with citrullinated arginine in collagen III. Bottom, schematic illustration of citrullinated integrin binding sites in collagen III. Integrin binding sites are labelled red. (**c**) Tandem MS spectra matching to a tryptic peptide containing the integrin binding motif NGR in fibronectin. Top left spectrum, a citrullinated form of the peptide from a synovial fluid sample. Bottom left spectrum, a citrullinated form of the peptide from *in vitro* citrullinated fibronectin. Top right spectrum, a non-citrullinated form of the peptide containing a deamidated asparagine residue from a synovial sample. Bottom right spectrum, a non-citrullinated form of the peptide containing a deamidated asparagine residue from an untreated fibronectin sample. cit, citrulline; c, carboxymethylated cysteine. O, hydroxyproline; n, deamidated asparagine. Bottom, schematic illustration of citrullinated integrin binding sites in fibronectin. Integrin binding sites are labelled red.

Among the citrullinated arginines there were three residues with a putative role in integrin–ECM interaction, namely R572 (RGD) in fibrinogen (Fig. 4a), R285 (GAOGER) in collagen α1(III) chain (Fig. 4b) and R234 (isoDGR/NGR) in FN (Fig. 4c). Integrin binding sites were selected for the further validation of the consequences of extracellular citrullination.

NGR motif in FN-I repeat 5 (FN-I5) spontaneously undergoes a gain-of-function conversion to isoDGR and is a well-known integrin binding site^12^ along with the RGD motif, which is present in FN-III repeat 10 (FN-III10)^13^. Interestingly, we could not detect the citrullination of the RGD site in synovial fluid. R234 in FN was found to be one of the most frequently citrullinated residues (detected in more than 40% of samples). The detection method for this modification was validated by citrullinating plasma FN enzymatically *in vitro*. This produced a spectrum similar to that seen in human synovial fluid derived samples (Fig. 4c). The spectrum was not identified in the untreated sample. Deamidation of N232 to isoD could also be seen both in synovial fluid and *in vitro*.

**Figure 5:**
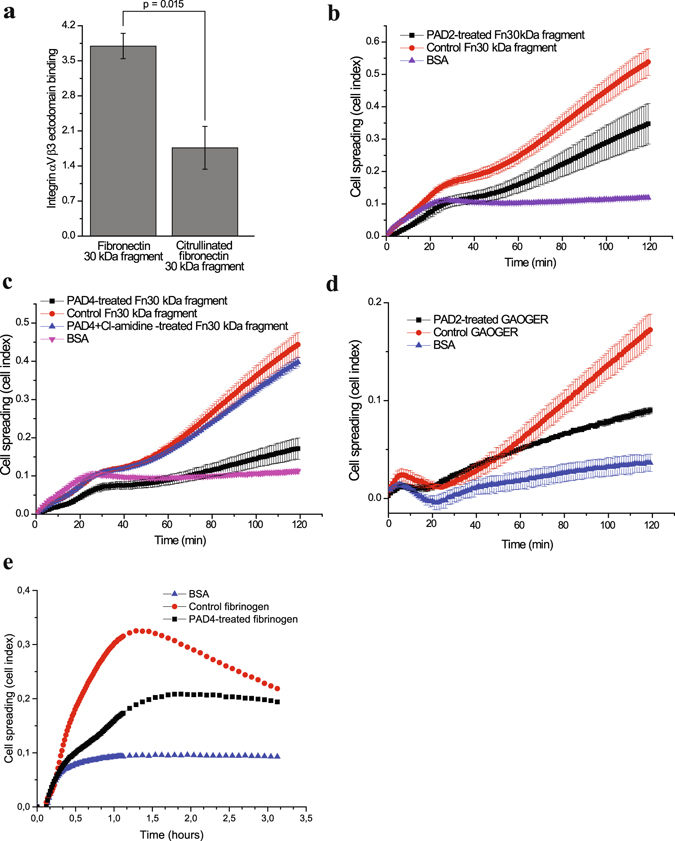
Citrullination inhibits integrin binding to isoDGR in fibronectin N-terminus and to GAOGER in collagen. (**a**) The binding of integrin αVβ3 ectodomain to citrullinated or control fibronectin N-terminal 30 kDa fragment. P-value between fibronectin (n = 3) and citrullinated fibronectin (n = 5) was calculated with two-tailed Student’s t-test from the mean values of three individual experiment normalized by background signal (BSA). Error bars show SEM. (**b**) Attachment and spreading of fibroblast like synoviocytes to PAD2-treated fibronectin N-terminal 30 kDa fragment real-time monitored by xCELLigence technology. BSA was the background control. Values are means ± SD of 3 parallel measurements from a single experiment. (**c**) Attachment and spreading of fibroblast like synoviocytes to PAD4-treated, control-treated (no enzyme) and PAD4 with inhibitor (Cl-amidine) –treated fibronectin N-terminal 30 kDa fragment real-time monitored by xCELLigence technology. BSA was the background control. Values are means ± SD of 3 parallel measurements from a single experiment. (**d**) Attachment and spreading of fibroblast like synoviocytes to PAD2-treated GAOGER assayed as above. (**e**) Attachment and spreading of fibroblast like synoviocytes to PAD4-treated fibrinogen assayed as above. Values are means ± SD of 3 parallel measurements from a single experiment.

To further study the effect of citrullination on integrin function, we treated the FN 30 kDa N-terminal fragment *in vitro* by PAD2 as well as PAD4 and measured recombinant integrin ectodomain αVβ6 binding to citrullinated and control FN (Fig. 5a). Integrin binding was reduced by more than 50% (p = 0.015) to the citrullinated FN fragment. In addition, fibroblast-like synoviocytes showed weaker adhesion to both the PAD4 and PAD2 treated 30 kDa N-terminal fragments when compared to the control; as was detected in xCELLigence real-time measurements (Fig. 5b,c). We could not exclude the possibility that citrullination of some other arginine from the N-terminal fragment could affect integrin binding and cell adhesion. However, isoDGR is the only site in the FN 30 kDa N-terminal fragment known to be recognized by integrins. The effect of citrullination by PAD2 on a triple helical GAOGER motif, a low affinity collagen receptor site^14^, was also tested and the reduction in cell adhesion was evident (Fig. 5d). The structural interpretation of citrullination related effects on GER-type collagen receptor binding site *in vitro* has been described earlier^15^. Finally, the treatment of fibrinogen by PAD4 also reduced the adhesion of synoviocytes (Fig. 5e).

**Figure 6:**
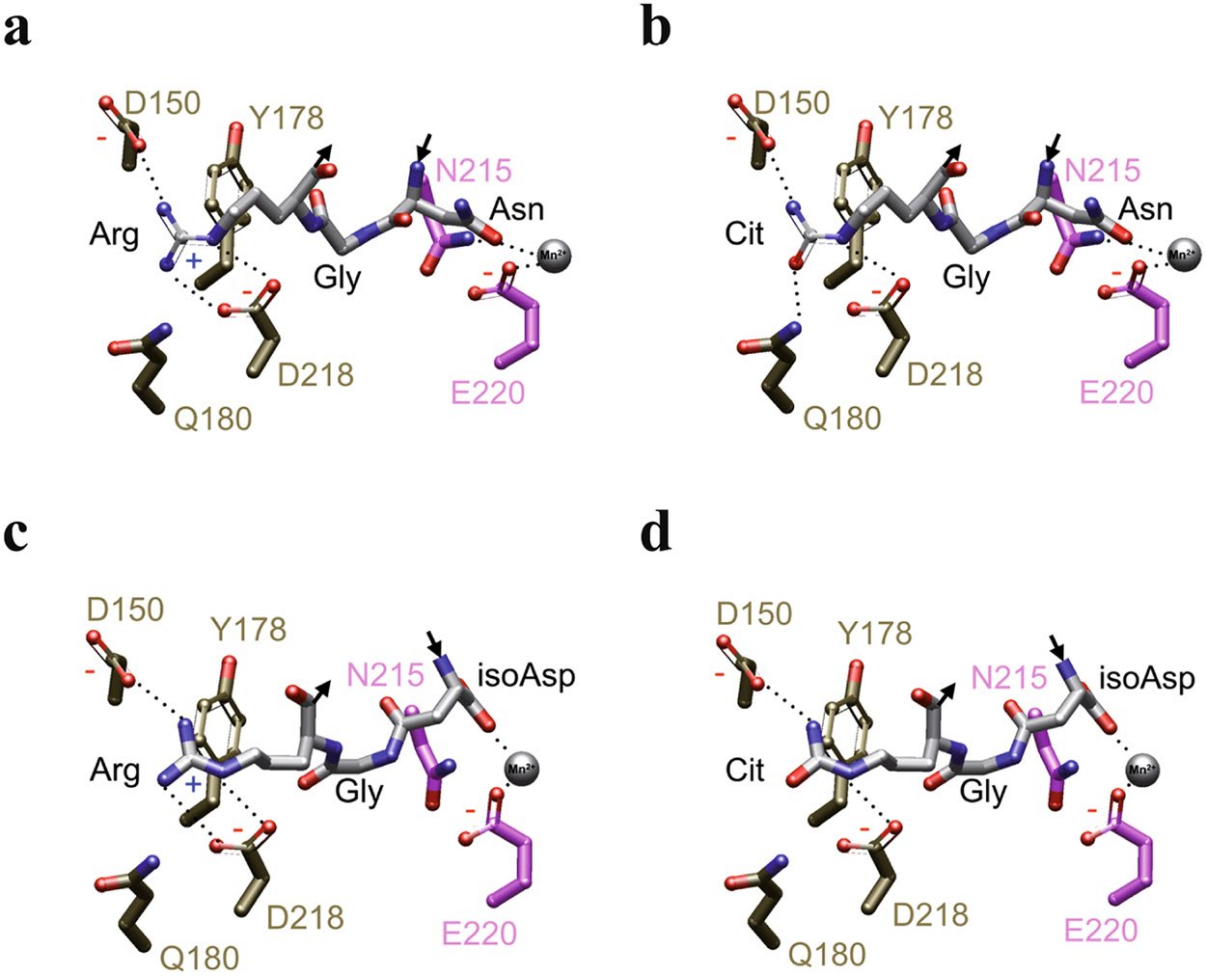
Citrullination inhibits integrin binding to isoDGR in fibronectin N-terminus. (**a**) Model of NGR at the binding site of αVβ3. (**b**) Model of NGcit at the binding site of αVβ3. (**c**) Model of isoDGR at the binding site of αVβ3. (**d**) Model of isoDGcit at the binding site of αVβ3. (**d**–**g**) NGR, NGcit, isoDGR and isoDGcit: white carbon atoms; Residues from αV: gold carbon atoms; Residues from β3; pink carbon atoms; metal cation: silver sphere; potential hydrogen bonds: dotted lines.

We also modelled the NGR and isoDGR motifs, placed them within the context of the αVβ3 binding site, and analysed the likely changes in interactions that would occur on conversion of the arginine to citrulline (Fig. 6). NGR motif spontaneously undergoes conversion to isoDGR in which the main-chain carbonyl becomes a short carboxylate side chain and the amide side chain of asparagine is incorporated into the main chain of FN-I5^12^. While isoDGR can bind to integrin αVβ3, NGR cannot^12, 16^. The binding of isoDGR to integrin αVβ3 is in the reverse main-chain direction in comparison to the binding of the classical RGD motif^17^, with arginine residues in either case interacting with an aspartate in the β-propeller domain of the integrin αV subunit, and isoAsp or aspartate interacting with the positively-charged metal cation at the metal ion dependent adhesion site, MIDAS, of the βI-like domain of the integrin β3 subunit. NGR binding likely fails because asparagine lacks the negative charge for interaction with the positively-charged metal cation and this appears to be a necessary feature for binding (Fig. 6a and c). NGR and hence isoDGR are surrounded by two glycine residues, i.e. GNGRG, that provide substantial flexibility to the main chain and would help it to adapt to the unusual main chain direction within the binding pocket at integrin αVβ3. However, the backbone of NGR is still less flexible than isoDGR because, as part of the rearrangement in forming isoDGR, a CH_2_ group from the original asparagine side chain is now part of the main chain of the pentapeptide. Together with the central glycine and the two peripheral glycine residues, this additional pivot point provides isoDGR with maximal flexibility to adapt its main chain to the requirements of the binding cavity.

With citrullination (Fig. 6b and d), a critical change occurs in the interactions between arginine of the peptide and the β-propeller domain of integrin αVβ3: the strong electrostatic interaction between arginine and the negative charge of D150 of the αV subunit is eliminated and this change alone is likely sufficient to markedly reduce or eliminate binding of the isoDGcit peptide to the integrin. If NGR could indeed bind to the integrin, which looks not to be the case, the loss of a second strong interaction would remove any possibility of binding (Fig. 6b).

## Discussion

Citrullination of extracellular proteins leads to the generation of new antigenic epitopes in inflammatory diseases, but very little is known about its effects on functional sites in proteins. Furthermore, the few published studied have focused on the consequences of *in vitro* enzyme treatments in purified proteins. Here we have mapped the citrullination sites in extracellular proteins using human synovial fluid samples derived form patients suffering from various rheumatoid diseases. More than 20% of the arginine residues that were identified as targets of extracellular citrullination, have a potential role in protein–protein interactions. Importantly, many of these functions are also essential in inflammation and tissue remodelling. For example, the replacement of R66 with alanine dramatically reduces the angiogenic potency of angiogenin^18^.

Integrin recognition motifs in ECM proteins are among the sites that are citrullinated by PADs *in vitro* ^15, 19^. Here, our observations indicate for the first time that integrin binding sites are citrullinated with high frequency in synovial fluid in chronic inflammation. *In vitro* citrullinated FN and collagen II have been shown to affect the behaviour of fibroblast like synoviocytes^15, 19^. Interestingly, the effect of FN citrullination on cell adhesion was considered to be independent of RGD, the classical integrin recognition motif^19^. In *in vitro* conditions the RGD of plasma FN was not deiminated even in the high enzyme concentration. Importantly, R234 (in NGR or isoDGR) located within the N-terminal part of FN was among the most frequently citrullinated arginine residues in inflamed synovial fluid and *in vitro*. The citrullination of R234 also prevented integrin binding. The role of the isoDRG site of FN I-5 is not completely understood but it has been suggested to participate in fibrillogenesis and ECM assembly^20, 21^. Thus, the intensive citrullination of FN R234 may affect the extracellular matrix re-assembly during the inflammatory process. In fibrinogen α-chain a known integrin binding site, RGD (R572), was detected to be citrullinated in synovial fluid. In addition, we report the arginine deimination in GAOGER site of collagen III.

The citrullination of extracellular proteins has been associated with inflammation in general, but disease-specificity has also been suggested^22–26^. Our data show that citrullination is a common inflammation related PTM that can be observed in synovial fluid samples derived from patients suffering from seropositive or seronegative RA, juvenile idiopathic arthritis, psoriatic arthritis, gout, or ankylosing spondylitis. Furthermore, no obvious quantitative or qualitative differences could be found between ACPA-positive and other joint inflammatory diseases. Given the facts, that (i) the general level of extracellular citrullination correlated with leukocyte infiltration into the joint (Fig. 3)^27^ and that (ii) extracellular citrullination seemed to be nonselective, it is unlikely that extracellular citrullination could be a diagnosis-specific process. Thus, the generation of highly specific ACPAs in RA cannot be explained by differences in extracellular citrullination but other mechanisms are needed to trigger the process. The fact that the inhibition of PAD4 in animal models decreases the amount of ACPAs^28^ is not contradictory with this idea. Similarly, the documented differences in citrullination between RA and osteoarthritis are likely caused by more aggressive inflammation and more prominent leukocyte infiltration into the joint in RA^23, 24, 29^. Still, intracellular citrullination may differ in distinct diseases^30^. Furthermore, the disease-dependent differences in the expression or proteolysis of extracellular proteins may generate new, disease-specific targets for PADs^31^.

During the last decade small molecule inhibitors against PAD2 and PAD4, such as N‐α‐benzoyl‐N5‐(2‐chloro‐1‐iminoethyl)‐L‐ornithine amide (Cl‐amidine) and GSK199, have been shown to reduce joint inflammation in collagen induced arthritis^28, 32^. Our findings may partially explain, how the PAD inhibitors block the vicious circles in rheumatoid disease.

We could not find support for a previous proposal^11^ that the residue in the N-2 position could determine the specificity. Our data suggest that there are no structural or sequential motifs dictating citrullination, only inherent solvent exposure or solvent exposure resulting from conformational changes, proteolytic cleavage or other process. Recently, it has been shown that the activity of PAD4 is regulated by direct interaction between the enzyme and PTPN22, protein tyrosine phosphatase non-receptor type 22^33^. In addition, some PAD4 autoantibodies can bind and regulate PAD4 activity by modulating calcium sensitivity^34^.

To conclude, our data indicate that citrullination is a common inflammation dependent modification in extracellular proteins. Importantly, it also affects arginine residues that are critical for protein function. Thus, PAD enzymes have the potency to affect the critical extracellular protein interactions that regulate immune response as well as tissue destruction and remodelling in joint inflammation.

## Methods

### Synovial fluid collection and clinical analysis

Synovial fluid (SF) samples were collected in the Turku University Hospital (permission number of the ethical committee, ETMK 15/1801/2013. Informed consent was obtained from all participants). All the methods used in this study were carried out in accordance with the relevant guidelines and regulations. The SF samples were placed on ice after puncture, centrifuged (10 min, +4 °C, 2500 g) and the supernatant and pellet were frozen separately (−80 °C). The supernatants were used for further analysis of citrullination. For the determining of the WBC and granulocyte percentage, SF samples were drawn into lithium-heparin tubes and the analysis was performed in the laboratory of the Turku University Hospital using manual chamber counting (sy-Leuk, sy-Diffi). Anti-citrullinated protein antibodies were analysed in the serum and in the SFs by the laboratory of the Turku University Hospital as described earlier^35, 36^. The diagnoses of the patients were set by rheumatologists based on the international classifications (ICD-10). (Supplementary Table 5).

### Mass spectrometry and data analysis

SF samples were diluted Tris-buffer (25% synovial fluid, 10 mM Tris-HCl pH 7.4) and heparin agarose were added (4%). After incubation o/n in gentle rotation (+4 °C), heparin agarose was washed intensively and heparin agarose –bound proteins were eluted with sodium chloride (1.5 M NaCl in 10 mM Tris-HCl pH 7.4). Albumin depletion was done as described earlier^37^. *In vitro* citrullination of fibronectin was performed in solution by adding PAD2 (purchased from Sigma-Aldrich, St. Louis, Missouri United States) or PAD4 (recombinant PAD4-GST, produced and purified as described earlier^15^ to citrullination buffer (40 mM Tris-HCl pH 7.4, 5 mM CaCl_2_). SF and FN samples with or without PAD treatment were digested with trypsin and the peptides were analysed by a nanoflow HPLC system (Easy-nLC1000, Thermo Fisher Scientific, Bremen, Germany) coupled to the Q Exactive mass spectrometer (Thermo Fisher Scientific).

Tandem mass spectra were searched against Human UniProtKB sequences with Proteome Discoverer software, version 1.4. (Thermo Fischer Scientific) using Mascot search engine (Matrix Science, version 2.4). Three separate searches of the tandem mass spectra were performed covering the following dynamic modifications: citrullination (R), deamidation (N, Q), oxidation (M), hydroxylation (K, P), phosphorylation (S, T, Y), carbamylation (K, peptide N-terminus), acetylation (K, protein N-terminus) and methylation (K, R). For all searches, maximum of two missed cleavages were allowed. At least two peptide identifications were required for protein identification.

To minimize the number of false positives among the PSMs of citrullinated peptides, the spectra leading to identification of citrullinated peptide (excluding those with C-terminal citrulline) were manually examined with Proteome Discoverer software. Only the citrullinations that were visible in the b or y fragment ion or in the subsequent b or y ion series were accepted. The citrullinations that could not be reliably distinguished from deamidations of N or Q were omitted. Citrullination in SF samples was quantified by counting the number of unique spectra matching to citrullinated peptide and comparing that to the total number of spectra leading to protein identification. Similarly, citrullination of a particular peptide or protein was quantified by comparing the number of spectra matching the citrullinated peptide(s) to the total number of spectra matching to the corresponding peptide or protein. The protein was classified as extracellular if its subcellular location was “secreted” or it belonged to the cellular component gene ontology term “extracellular region”, according to the UniProt entry of the protein. The proteins that form variable regions of immunoglobulins were excluded from further analysis.

GO Slimmer Tool (http://amigo1.geneontology.org/cgi-bin/amigo/slimmer) of AmiGO web application, version 1.8^38^ was used to map the citrullinated proteins to selected GO slim terms, and the mappings were visualized using Cytoscape version 3.4.0^39^.

### PAD activity assay

PAD activity of synovial fluids, PAD2 and PAD4 was measured with an assay of fibrinogen citrullination as described earlier^40^. Briefly, wells of 96-well plate wells were coated o/n with fibrinogen in PBS (0.1 mg/ml) o/n at +4 °C and blocked with BSA (1 mg/ml) 1 h at RT. The samples were diluted 1/4 with citrullination buffer (40 mM Tris-HCl, 2 mM or 5 mM CaCl_2_, pH 7.4) and incubated on wells o/n at +37 °C. The long incubation time was used for reaching maximal citrullination but also shorter incubations times can be used. The amount of citrullinated fibrinogen was measured using anti-citrullinated fibrinogen antibody (Mouse Anti-Citrullinated Fibrinogen (20B2), ModiQuest; 1 h, RT) and Eu-labeled anti-mouse secondary antibody (PerkinElmer, Waltham, Massachusetts; 1 h, RT). The fluorescence was measured from enhancer filled wells by Victor3 multilabel counter (PerkinElmer, Waltham, Massachusetts). Wells were washed three times with PBS after each step.

### Structural analysis

Protein structures were obtained from the Protein Data Bank (PDB)^41^. Structures were visualized and figures of structures prepared using Bodil^42^. Sequences surrounding arginine residues found to be citrullinated were obtained from the structure file when available, and from the UniProt database^43^.

The peptides NGR and isoDGR were built using Maestro 2D sketcher, prepared with LigPrep and mutated as necessary using Bodil. Integrin αVβ3 (PDB ID: 1L5G)^17^ was prepared as the receptor using the Protein Preparation Wizard panel (maestro version-10.3.015 Schrödinger suit) by assigning bond order, correcting ionization states and creating disulphide bonds. The most probable ionization state of the MIDAS metal ion was predicted using water as the solvent at pH 7 ± 3.0. Both NGR and isoDGR sequence motifs were placed within the context of the integrin αVβ3 binding site and bound RGD peptide (PDB ID: 1L5G) by using the Bodil software package followed by energy minimization of the complex using the OPLS-2005 force field in maestro version-10.3.015 Schrödinger suit for optimization of bond lengths and angles, including torsion angles. Acceptable side-chain torsion angles were also explored using Bodil.

### Protein binding assay

Plates (96-well DNA-BIND; Corning Costar, Corning, NY, USA) were coated o/n with FN N-terminal 30 kDa fragment (3 µg/cm^3^, purchased from Sigma-Aldrich, St. Louis, Missouri United States) or BSA (1 mg/ml) in +4 °C. The wells were blocked with BSA (1 mg/ml, RT, 1 h). After citrullination by PAD2 or PAD4 (2.4 U/ml, o/n at +37 °C), the wells were washed, and a recombinant integrin αVβ3 (human, R&D Biosystems; 5 µg/ml) was allowed to bind for 1 h at room temperature in Delfia assay buffer (PerkinElmer, Waltham, Massachusetts). Anti-αV antibody (L230, ATCC) was used as a primary antibody (1 h, RT, 1/1000 dilution) and Eu-labeled anti-mouse antibody (PerkinElmer, Waltham, Massachusetts) as a secondary antibody (1 h, RT, 1/1000 dilution). The wells were washed and enhancement solution added. Signal was detected with time-resolved fluorescence spectrophotometry (Victor3 multilabel counter; PerkinElmer, Waltham, Massachusetts). The washing steps were always three times with PBS.

### Real-time spreading assay (xCELLigence)

Primary fibroblast like synoviocytes (the kind gift of the late Professor Yrjö Konttinen, University of Helsinki, Finland)^15^ were cultured in DMEM with 10% FCS for maintaining. Plates (96-well E-Plate 96; ACEA Biosciences, San Diego, CA, USA were coated o/n with FN N-terminal 30 kDa fragment or fibronectin from bovine plasma (3 µg/cm^3^, Sigma-Aldrich, St. Louis, Missouri United States), fibrinogen (100 µg/ml), synthesized triple helical collagen mimetic peptide GPC-(GPP)5 -GAOGER-(GPP)5-GPC-NH_2_ (10 µg/ml) or BSA (1 mg/ml) in +4 °C. The wells were blocked with BSA (1 mg/ml, RT, 1 h) and treated with PAD2 or PAD4 (1.2 U/ml, 50–150 µg/ml), 2 h - o/n, +37 °C), or PAD4 supplemented with Cl-amidine (500 µM; N-α-benzoyl-N5-(2-chloro-1-iminoethyl)-L-Ornamide; Merck MilliPore) in 40 mM Tris, 5 mM CaCl_2_, pH 7.4. Detached fibroblast like synoviocytes (trypsinised; P5-7 were used in the experiments; 15 000–20 000 cells per well) were seeded to the wells in serum-free DMEM. Cell spreading was measured with the xCELLigence system (Roche Applied Science).

### Data availability

The mass spectrometry proteomics data along with a detailed description of the methods have been deposited to the ProteomeXchange Consortium^44^ via the PRIDE partner repository with the dataset identifier PXD004017. Other data generated or analysed during this study are included in this published article (and its supplementary information files).

## Electronic supplementary material

Supplementary information
