# Supplementary material for: Joint inflammation related citrullination of functional arginines in extracellular proteins
Source: Sci Rep. 2017 Aug 15;7:8246. doi: 10.1038/s41598-017-08597-4 (PMC5557964; doi:10.1038/s41598-017-08597-4)
Supplement: Supplementary file 1 — Supplementary information [file 41598_2017_8597_MOESM1_ESM.pdf]

Joint inflammation related citrullination of functional arginines in extracellular proteins

Kalle H. Sipilä,[1] Vipin Ranga,[2] Pekka Rappu,[1] Markku Mali,[3] Laura Pirilä,[3] Ilona Heino,[1] Johanna Jokinen,[1] Jarmo Käpylä,[1] Mark S. Johnson[2] & Jyrki Heino[1,*]

[1]Department of Biochemistry, University of Turku, Turku, Finland. [2]Structural Bioinformatics Laboratory, Biochemistry, Faculty of Science and Engineering, Åbo Akademi University, Turku, Finland. [3]Turku University Hospital, Division of Medicine, Department of Rheumatology, and University of Turku, Turku, Finland.

Supplementary information

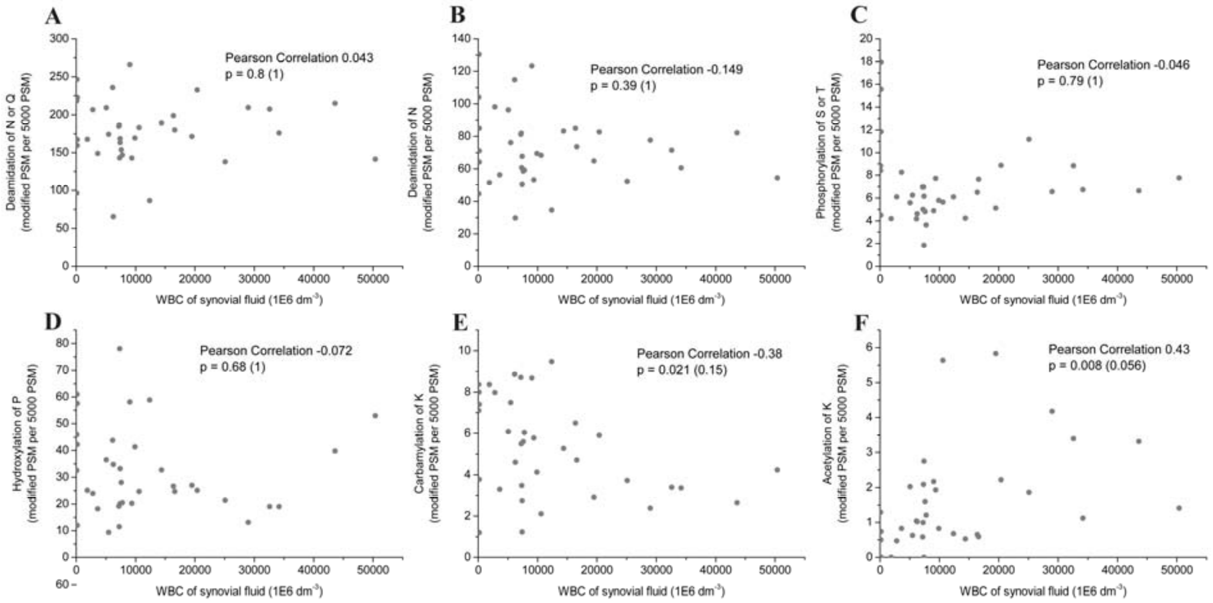

Supplementary Figure 1. The global analysis of deamidation (A-B), phosphorylation (C), hydroxylation (D), carbamylation (E) and acetylation (F). The analysis was performed by LC-MS/MS of heparin-agarose fractionated synovial fluids collected from joint inflammatory patients (n = 40). Peptide-spectrum-matches (PSM) were normalized to 5000 PSM per sample. The number of PTM-containing PSM was correlated to white blood count of synovial fluid (WBC). The Bonferroni-corrected p-values are in parenthesis.

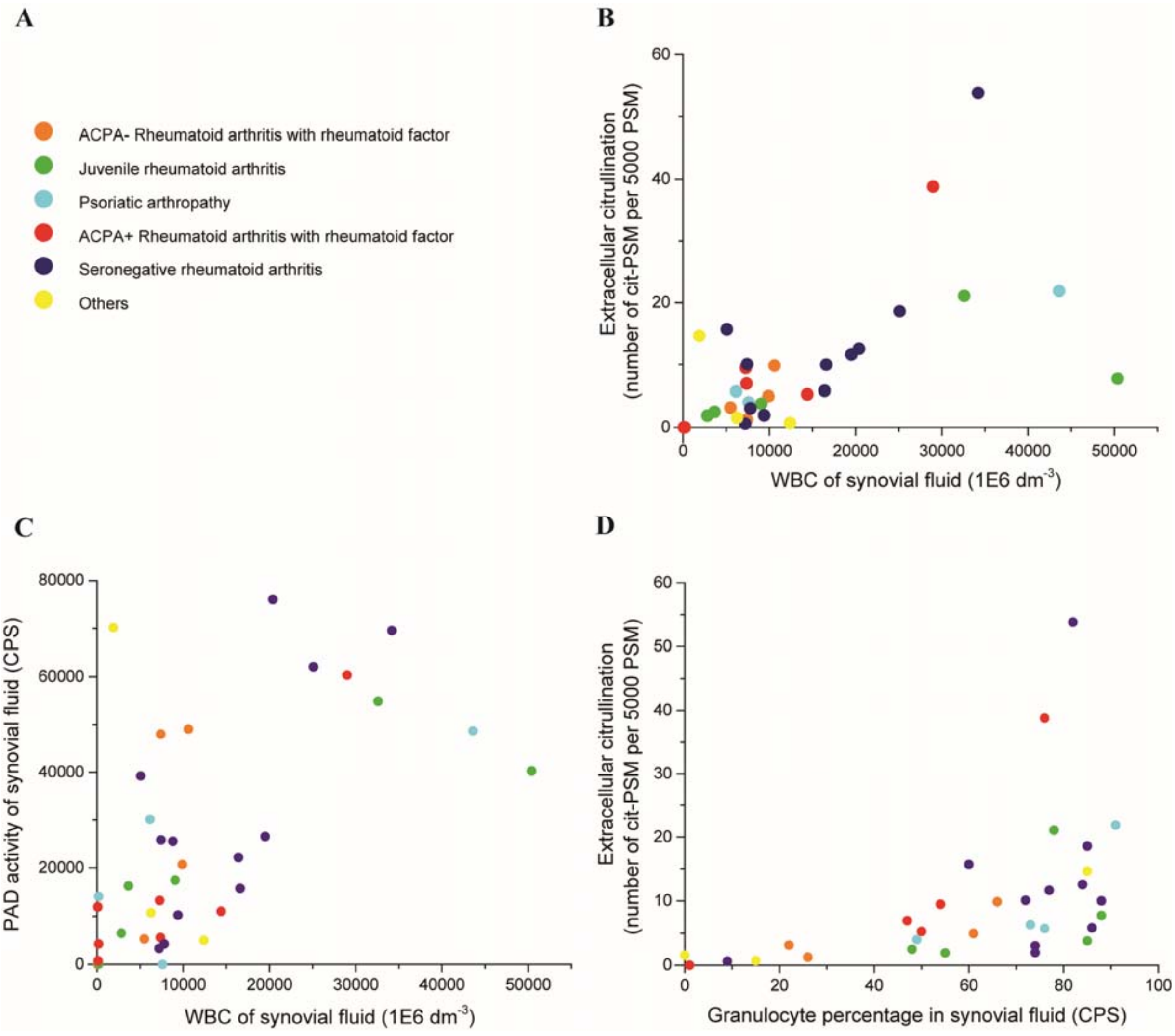

Supplementary Figure 2. Extracellular citrullination in synovial fluid does not show obvious differences between clinical diagnoses. **(**A) Classification of the patients by 8 different clinical diagnoses (RF = rheumatoid factor, ACPA anti-citrullinated protein antibodies). (B-D) Extracellular citrullination or PAD activity of synovial fluid shown as a function of white blood count (WBC) or granulocyte percentage.

Supplementary Table 1. Citrullinated proteins and arginine residues *in vivo*.

Function	Gene Symbol	Protein name	Citrullination site (mature peptide)
Immune system	C3	Complement C3	R682
	CFH	Complement factor H	R1192
	IGHM	Ig mu chain C region	R120
Growth factors	ANG	Angiogenin	R66
	MST1	Hepatocyte growth factor-like protein	R465
	KNG1	Kininogen-1	R31<br>R374<br>R439
Cell adhesion and extracellular matrix	COL3A1	Collagen III	R285
	COMP	Cartilage oligomeric matrix protein	R227
	EFEMP1	EGF-containing fibulin-like extracellular matrix protein	R131
	FBN1	Fibrillin-1	R18
	FN1	Fibronectin	R234<br>R409<br>R1131<br>R1542<br>R2325
	FGA	Fibrinogen alpha chain	R104<br>R110<br>R252<br>R491<br>R554<br>R572<br>R602
	MXRA5	Matrix-remodeling-associated protein 5	R736
	PAPLN	Papilin	R824
	PCOLCE	Procollagen C-endopeptidase enhancer 1	R418
	PRG4	Proteoglycan 4	R1367<br>R1276<br>R1293<br>R1300<br>R56<br>R168
	RARRES2	Retinoic acid receptor responder protein 2	R105
Protease inhibitors and proteases	CTSG	Cathepsin G	R64

			R74<br>R136<br>R136 and R142<br>R150 and R152<br>R165 and R172<br>R172<br>R212
	ITIH4	Inter-alpha-trypsin inhibitor heavy chain H4	R617<br>R621
	ELANE	Neutrophil elastase	R117
	F2	Prothrombin	R437
	PLG	Plasminogen	R70
	AMBP	Protein AMBP	R121<br>R166<br>R278
Others	ALB	Albumin	R87<br>R263
	APOA4	Apolipoprotein A-IV	R259
	APOE	Apolipoprotein E	R167<br>R172<br>R180<br>R189<br>R213

Supplementary Table 2. *In vivo* citrullinated arginine residues with known function.

Citrullination site (mature peptide)	Protein name	Function of the arginine	Reference
R66	Angiogenin	Needed for angiogenic activity	Shapiro and Vallee (1992)
R172	Apolipoprotein E	Needed for ApoE binding to LDL receptor	Morrow et al. (2000)
R136	Cathepsin G	Needed for bactericidal activity	Shafer et al. (1996)
R285	Collagen III	Binding site of collagen receptor integrins, "GAOGER"	Siljander et al. (2004)
R1192	Complement factor H	Mutation causes age-related macular degeneration and haemolytic uraemic syndrome	Carioli et al. (2001), Perkins and Goodship (2002), Raychaudhuri et al. (2012)
R104	Fibrinogen alpha chain	Plasmin cleavage site	UniProtKB - P02671 (FIBA_HUMAN)
R110		Mutation causes hypofibrinogenemia	Asselta et al. (2015)
R554		Mutation associates with abnormal fibrin polymerization and thrombophilia and amyloidosis	Benson et al. (1993), Koopman et al. (1993)
R572		Binding site of integrins, "RGD"	Plow et al. (1987)
R234	Fibronectin	Binding site of integrins, "isoDGR"	Curnis et al. (2006)
R465	Hepatocyte growth factor-like protein	Cleavage site for proteolytic activation	Ganesan et al. (2011)
R437	Prothrombin	Mutation impairs the fibrinogen clotting activity	Miyata et al. (1987)

Supplementary Table 3. Structural analysis of citrullination sites *in vivo*.

Uniprot ID	Protein	Sequence*	Extended sequence	arginine residue position in PDB file	PDB ID/Chain	Side chain orientation	Secondary structure	N-2 (Size)
P03950	Angiogenin	AIcENKNGNPH#ENLR	AIcENKNGNPH#ENLR	66	1H52/A	exposed	beta turn	Pro (Small)
P02649	Apolipoprotein E	AQAWGE#LR	AQAWGE#LRAR	213	2L7B/A (NMR)	exposed	alpha helix	Gly (Small)
P02649	Apolipoprotein E	E#LGPLVEQGR	AIRE#LGPLVEQGR	180	2L7B/A (NMR)	exposed	alpha helix	Arg (Large)
P02649	Apolipoprotein E	EGAE#GLSAIR	EGAE#GLSAIR	172	2L7B/A (NMR)	exposed	alpha helix	Ala (Small)
P02649	Apolipoprotein E	LAVYQAGA#EGAER	LAVYQAGA#EGAER	167	2L7B/A (NMR)	exposed	loop	Gly (Small)
P02649	Apolipoprotein E	LGPLVEQG#VR	LGPLVEQG#VRAA	189	2L7B/A (NMR)	exposed	loop	Gln (Large)
P49747	Cartilage oligomeric matrix protein	FcPDGSPSEcHEHADcVLE#DGSR	FcPDGSPSEcHEHADcVLE#DGSR	247	3FBY/A	exposed	beta turn	Leu (Large)
P08311	Cathepsin G	#ENTQQHITAR	NIQR#ENTQQHITAR	76	1AU8/A	exposed	loop	Gln (Large)
P08311	Cathepsin G	#GTDTL#EVQLR	VSMR#GTDTL#EVQLR	148, 156	1AU8/A	exposed, partially exposed	loop, beta sheet	Met (Large), Thr (Small)
P08311	Cathepsin G	REnTQQHITA#R	REnTQQHITA#R	86	1AU8/A	exposed	beta sheet	Thr (Small)
P08311	Cathepsin G	SSGVPPEVFT#VSSFLPWIR	SSGVPPEVFT#VSSFLPWIR	230	1AU8/A	partially exposed	beta sheet	Phe (Large)
P08311	Cathepsin G	VQ#D#QcLR	LRVQ#D#QcLR	164, 166	1AU8/A	exposed, exposed	loop, alpha helix	Val (Large), Arg (Large)
P08311	Cathepsin G	VSm##GTDTLR	RVSm##GTDTLR	147, 148	1AU8/A	exposed, exposed	loop, loop	Ser (Small), Met (Large)
P02461	Collagen III	GAOGE#GROGLOGAAGAR	GAOGE#GROGLOGAAGAR					Gly (Small)
P01024	Complement C3	ENPM#FScQR	ENPM#FScQR	82	2A73/B	exposed	loop	Pro (Small)
P01024	Complement C3	SGQSED#QPVPGQQMTLK	SGQSED#QPVPGQQMTLK	551	2A73/A	exposed	loop	Glu (Large)

P08603	Complement factor H	IVSSAMEPD#EYHFGQAVR	IVSSAMEPD#EYHFGQAVR	148	2WII/C	exposed	loop	Pro (Small)
P08603	Complement factor H	LSS#SHTLR	RLSS#SHTLR	1210	3R62/A	exposed	loop	Ser (Small)
Q12805	EGF-containing fibulin-like extracellular matrix protein	NPADPQ#IPSNPSHR	NPADPQ#IPSNPSHR					Pro (Small)
P35555	Fibrillin-1	#GGGGHDALKGPNVcGSR	RAKR#GGGGHDALKGPNVcGSR	45	2M74/A (NMR)	exposed	loop	Lys (Large)
P02671	Fibrinogen alpha chain	DNTYN#VSEDLR	DNTYN#VSEDLR	110	3GHG/A	exposed	alpha helix	Tyr (Large)
P02671	Fibrinogen alpha chain	ESSSHHPGIAEFPS#GK	ESSSHHPGIAEFPS#GKSS		3GHG/A	missing, C-terminal		Pro (Small)
P02671	Fibrinogen alpha chain	EVVTSEDGSDcPEAMDLGTLSGIGTLDGF#HR	EVVTSEDGSDcPEAMDLGTLSGIGTLDGF#HRHP		3GHG/A	missing, C-terminal		Gly (Small)
P02671	Fibrinogen alpha chain	GDFSSANN#DNTYNR	GDFSSANN#DNTYNR	104	3GHG/A	exposed	alpha helix	Asn (Small)
P02671	Fibrinogen alpha chain	MADEAGSEADHEGTHSTK#GHAK	MADEAGSEADHEGTHSTK#GHAK					Thr (Small)
P02671	Fibrinogen alpha chain	MELERPGGNEIT#GGSTSYGTGSETESPR	MELERPGGNEIT#GGSTSYGTGSETESPR		3GHG/A	missing		Ile (Large)
P02671	Fibrinogen alpha chain	QFTSSTSYN#GDSTFESK	QFTSSTSYN#GDSTFESK					Tyr (Large)
P02751	Fibronectin	GNLLQcIcTGNG#GEWK	GNLLQcIcTGNG#GEWK	234	3EJH/A	exposed	beta sheet	Asn (Small)
P02751	Fibronectin	RPGGEPSPEGTTGQSYNQYSQ#YHQR	RPGGEPSPEGTTGQSYNQYSQ#YHQR					Ser (Small)
P02751	Fibronectin	WLPSSSPVTGY#VTTTPK	WLPSSSPVTGY#VTTTPK					Gly (Small)
P02751	Fibronectin	DGQE#DAPIVNK	DGQE#DAPIVNK					Gln (Large)
P02751	Fibronectin	#DNmKWcGTTQNYDADQK	SEGR#DNmKWcGTTQNYDADQK	440	3M7P/A	exposed	loop	Gly (Small)

P26927	Hepatocyte growth factor-like protein	cGSEAQP#QEATTVScFR	cGSEAQP#QEATTVScFR					Gln (Large)
P26927	Hepatocyte growth factor-like protein	L#VVGGHPGNSPWTVSLR	RSKL#VVGGHPGNSPWTVSLR	483	4QT8/C	exposed	loop	Lys (Large)
P01871	Ig mu chain C region	VSVFVPP#DGFFGNPR	VSVFVPP#DGFFGNPR					Pro (Small)
Q14624	Inter-alpha-trypsin inhibitor heavy chain H4	#GWNRQAGAAGSR	FSPR#GWNRQAGAAGSR					Pro (Small)
Q14624	Inter-alpha-trypsin inhibitor heavy chain H4	RGWN#QAGAAGSR	RGWN#QAGAAGSR					Trp (Large)
P01042	Kininogen-1	DQGHGHQ#GHGLGHGHEQQHGLGHGHK	DQGHGHQ#GHGLGHGHEQQHGLGHGHK					His (Large)
P01042	Kininogen-1	HE#DQGHGHQ#GHGLGHGHEQQHGLGHGHK	HKHE#DQGHGHQ#GHGLGHGHEQQHGLGHGHK					His (Large)
Q9NR99	Matrix-remodeling-associated protein 5	HSEKEPETNVAEG#R	HSEKEPETNVAEG#RVFE					Glu (Large)
P08246	Neutrophil elastase	#LGNGVQcLAMGWGLLGR	AQGR#LGNGVQcLAMGWGLLGR	129	2Z7F/E	exposed	loop	Gly (Small)
O95428	Papilin	STHTDGGGSSPSGEQEPSQH#TGAAVQR	STHTDGGGSSPSGEQEPSQH#TGAAVQR					Gln (Large)
P00747	Plasminogen	M#DVVLFEK	IIRM#DVVLFEK	70	4DUR/A	solvent exposed	beta sheet	Arg (Large)
Q15113	Procollagen C-endopeptidase enhancer 1	KcPSQPV#AAASQD	KcPSQPV#AAASQD		1UAP/A	missing, C-terminal		Pro (Small)
P02760	Protein AMBP	FS#HHGPTITAK	KKFS#HHGPTITAK	121	3QKG/A	exposed	loop	Phe (Large)
P02760	Protein AMBP	GPc#AFIQLWAFDAVK	RGPc#AFIQLWAFDAVK	92	1BIK/A	exposed	loop	Pro (Small)
Q92954	Proteoglycan 4	AIGPSQTHTI#IQYSPAR	AIGPSQTHTI#IQYSPAR					Thr (Small)
Q92954	Proteoglycan 4	AITT#SGQTLSK	AITT#SGQTLSK					Thr (Small)

Q92954	Proteoglycan 4	cFESFE#GR	cFESFE#G REC					Phe (Large)
Q92954	Proteoglycan 4	IQYSPA#LA YQDK	IQYSPA#L AYQDK					Pro (Small)
Q92954	Proteoglycan 4	NSAAN#EL QK	NSAAN#E LQK					Ala (Small)
Q92954	Proteoglycan 4	RPALNYPVY GETTQV#R	RPALNYP VYGETTQ V#RRRF					Gln (Large)
P00734	Prothrombin	ENLD#DIAL MK	ENLD#DI ALMK	418	4NZQ/A	partially exposed	loop	Leu (Large)
P00734	Prothrombin	G#VTGWG NLK	GYKG#VT GWGNLK	457	4NZQ/A	buried	beta sheet	Lys (Large)
Q99969	Retinoic acid receptor responder protein 2	LVHcPIETQ VL#EAEEH QETQcLR	LVHcPIET QVL#EAE EHQETQc LR					Val (Large)

* # denotes *in vivo* citrullinated arginine

Supplementary Table 4. Structural analysis of fibronectin citrullination *in vitro*.

Site	Sequence	PDB ID/Chain	arginine residue position in PDB file	Secondary Structure	Side chain orientation	N-2 (Size)
R2325	SQ#YH	No structure				Ser (Small)
R409	GR#DN	3M7P/A	440	loop	exposed	Gly (Small)
R206	DT#TS	2RKY/A	206	beta turn	exposed	Asp (Small)
R210	SY#IG	2RKY/A	210	beta strand C terminal	exposed	Ser (Small)
R206+R210	DT#TSY#IG	2RKY/A, 2RKY/A	206, 210	beta turn, beta strand C terminal	exposed, exposed	Asp (Small), Ser (Small)
R101	RG#IS	2CG6/A	101	beta strand N terminal	exposed	Arg (Large)
R519	QG#GR	3GXE/A	550	beta turn	exposed	Gln (Large)
R1369	SG#PR	3T1W/A	1491	loop	exposed	Ser (Small)
R1004	LT#RG	No structure				Leu (Large)
R1542	GY#VT	No structure				Gly (Small)
R2218	SS#WC	2EC3/A (NMR)	18	loop	exposed	Ser (Small)
R234	NG#GE	2RKY/A	234	beta turn	exposed	Asn (Small)
R1004+R1005	LT##GQ	No structure				Leu (Large), Thr (Small)
R1358	GY#IR	4LXO/A	1358	beta strand	partially exposed	Gly (Small)
R1369+R1371	SG#P#ED	4LXO/A	1369, 1371	loop, beta strand N terminal	exposed, exposed	Ser (Small), Arg (Large)
R197/R199	TS#N#CN	2RKY/A	197, 199	loop, beta strand N terminal	exposed, partially exposed	Thr (Small), Arg (Large)
R2213	HF#CD	2EC3/A (NMR)	13	loop	exposed	His (Large)
R1957	IG#KK	3R8Q/A	268	beta strand	exposed	Ile (Large)
R99	AG#GR	2CG6/A	99	beta turn	exposed	Ala (Small)
R613	IL#WR	2HA1/A (NMR)	36	beta strand	partially exposed	Ile (Large)
R124/R125	TW##PH	2CG6/A	124, 125	beta strand, beta strand	exposed, exposed	Thr (Small), Trp (Large)
R83	YE#PK	1O9A/A (NMR)	83	beta strand	exposed	Tyr (Large)
R83+R99	YE#PKDSMIWDCTCIGAG#GR	1O9A/A (NMR), 2CG6/A	83, 99	beta strand, beta turn	exposed, exposed	Tyr (Large), Ala (Small)

R613+R615	IL#W#PK	2HA1/A (NMR)	36, 38	beta strand, beta strand	partially exposed, partially exposed	Ile (Large), Arg (Large)
R221	DN#GN	2RKY/A	221	beta turn	exposed	Asp (Small)
R221+R234	DN#GNNLLQ CICTGNG#GE	2RKY/A	221, 234	beta turn, beta turn	exposed, exposed	Asp (Small), Asn (Small)
R241	CE#HT	2RKY/A	241	loop C terminal	partially exposed	Cys (Small)
R2295	GQ#GW	No structure				Gly (Small)
R2235	WD#QG	2EC3/A (NMR)	35	beta strand	exposed	Trp (Large)
R1131	QE#DA	No structure				Gln (Large)

Supplementary Table 5. Sample and patient information.

Sample Number	Gender	Age (at the moment of sampling)	Diagnosis		ACPA
2,17	Female	25	M08.4	Pauciarticular juvenile rheumatoid arthritis	-
3	Male	48	L40.5+M07.3	Arthropathic psoriasis + other psoriatic arthropathy	-
4,5,28,29	Female	53	M06.9 (M31.3)	Rheumatoid arthritis, unspecified (Wegener's granulomatosis)	-
6,19	Female	43	M05.8	Other rheumatoid arthritis with rheumatoid factor	-
7	Female	63	M05.8	Other rheumatoid arthritis with rheumatoid factor	+
8	Female	74	M05.8	Other rheumatoid arthritis with rheumatoid factor	-
9	Male	49	L40.5+M07.3	Arthropathic psoriasis + other psoriatic arthropathy	-
10	Male	53	M10.9	Gout, unspecified	-
11,16	Female	44	M06.8	Other specified rheumatoid arthritis, unspecified site	-
12	Female	22	M08.3	Juvenile rheumatoid polyarthritis (seronegative)	-
13	Female	51	M07.3	Other psoriatic arthropathy	-
14	Female	48	M06.0 (L40.5+M07.3)	Rheumatoid arthritis without rheumatoid factor, (Arthropathic psoriasis + other psoriatic arthropathies)	-
15	Female	63	M05.8	Other rheumatoid arthritis with rheumatoid factor	+
18	Female	50	M08.0 (M05.8)	Unspecified juvenile rheumatoid arthritis (Other rheumatoid arthritis with rheumatoid factor)	+
20	Female	65	M05.8	Other rheumatoid arthritis with rheumatoid factor	+
21	Female	48	M05.8	Other rheumatoid arthritis with rheumatoid factor	-
22	Female	66	M05.8	Other rheumatoid arthritis with rheumatoid factor	+
23,24	Female	61	M06.8	Other specified rheumatoid arthritis, unspecified site	-
25	Male	49	M06.8	Other specified rheumatoid arthritis, unspecified site	-

26	Female	19	M08.3	Juvenile rheumatoid polyarthritis (seronegative)	-
27	Female	65	M05.8	Other rheumatoid arthritis with rheumatoid factor	+
30	Female	64	M05.8	Other rheumatoid arthritis with rheumatoid factor	-
31	Female	23	M06.9	Rheumatoid arthritis, unspecified	-
32	Male	18	M08.3	Juvenile rheumatoid polyarthritis (seronegative)	-
33,40	Female	60	M06.0	Rheumatoid arthritis without rheumatoid factor	-
34	Male	43	M45	Ankylosing spondylitis	-
35	Male	62	M05.8	Other rheumatoid arthritis with rheumatoid factor	+
36	Male	24	M08.3	Juvenile rheumatoid polyarthritis (seronegative)	-
37	Female	49	L40.5+M07.3	Arthropathic psoriasis + other psoriatic arthropathy	-
38	Male	55	M13.9 L42	Unspecified arthritis, multiple sites+ Pityriasis rosea	-
39	Female	62	M05.8	Other rheumatoid arthritis with rheumatoid factor	+
41	Male	54	M06.0	Rheumatoid arthritis without rheumatoid factor	-

References

Asselta R, Platè M, Robusto M, Borhany M, Guella I, Soldà G, Afrasiabi A, Menegatti M, Shamsi T, Peyvandi F, Duga S (2014) Clinical and molecular characterisation of 21 patients affected by quantitative fibrinogen deficiency. *Thromb. Haemost.* 113**:** 567–576

Benson MD, Liepnieks J, Uemichi T, Wheeler G, Correa R (1993) Hereditary renal amyloidosis associated with a mutant fibrinogen α–chain. *Nat. Genet.* 3**:** 252–255

Caprioli J, Bettinaglio P, Zipfel PF, Amadei B, Daina E, Gamba S, Skerka C, Marziliano N, Remuzzi G, Noris M, Itaslian Registry of Familial and Recurrent HUS/TTP (2001) The molecular basis of familial hemolytic uremic syndrome: mutation analysis of factor H gene reveals a hot spot in short consensus repeat 20. *J. Am. Soc. Nephrol.* 12**:** 297–307

Curnis F, Longhi R, Crippa L, Cattaneo A, Dondossola E, Bachi A, Corti A (2006) Spontaneous formation of L-isoaspartate and gain of function in fibronectin. *J. Biol. Chem.* 281**:** 36466–36476

Ganesan R, Kolumam GA, Lin SJ, Xie M-H, Santell L, Wu TD, Lazarus RA, Chaudhuri A, Kirchhofer D (2011) Proteolytic Activation of Pro-Macrophage-Stimulating Protein by Hepsin. *Mol. Cancer Res.* 9**:** 1175–1186

Koopman J, Haverkate F, Grimbergen J, Lord ST, Mosesson MW, DiOrio JP, Siebenlist KS, Legrand C, Soria J, Soria C (1993) Molecular basis for fibrinogen Dusart (A alpha 554 Arg-->Cys) and its association with abnormal fibrin polymerization and thrombophilia. *J. Clin. Invest.* 91**:** 1637–1643

Miyata T, Morita T, Inomoto T, Kawauchi S, Shirakami A, Iwanaga S (1987) Prothrombin Tokushima, a replacement of arginine-418 by tryptophan that impairs the fibrinogen clotting activity of derived thrombin Tokushima. *Biochemistry* 26**:** 1117–1122

Morrow JA, Arnold KS, Dong J, Balestra ME, Innerarity TL, Weisgraber KH (2000) Effect of Arginine 172 on the Binding of Apolipoprotein E to the Low Density Lipoprotein Receptor. *J. Biol. Chem.* 275**:** 2576–2580

Perkins SJ, Goodship TH. (2002) Molecular modelling of the C-terminal domains of factor H of human complement: a correlation between haemolytic uraemic syndrome and a predicted heparin binding site. *J. Mol. Biol.* 316**:** 217–224

Plow EF, Pierschbacher MD, Ruoslahti E, Marguerie G, Ginsberg MH (1987) Arginyl-glycyl-aspartic acid sequences and fibrinogen binding to platelets. *Blood* 70**:** 110–115

Raychaudhuri S, Iartchouk O, Chin K, Tan PL, Tai AK, Ripke S, Gowrisankar S, Vemuri S, Montgomery K, Yu Y, Reynolds R, Zack DJ, Campochiaro B, Campochiaro P, Katsanis N, Daly MJ, Seddon JM (2011) A rare penetrant mutation in CFH confers high risk of age-related macular degeneration. *Nat. Genet.* 43**:** 1232–1236

Shafer WM, Hubalek F, Huang M, Pohl J (1996) Bactericidal activity of a synthetic peptide (CG 117-136) of human lysosomal cathepsin G is dependent on arginine content. *Infect. Immun.* 64**:** 4842–4845

Shapiro R, Vallee BL (1992) Identification of functional arginines in human angiogenin by site- directed mutagenesis. *Biochemistry* 31**:** 12477–12485

Siljander PR-M, Hamaia S, Peachey AR, Slatter DA, Smethurst PA, Ouwehand WH, Knight CG, Farndale RW (2004) Integrin activation state determines selectivity for novel recognition sites in fibrillar collagens. *J. Biol. Chem.* 279**:** 47763–47772
